# The association between excise tax structures and the price variability of alcoholic beverages in the United States

**DOI:** 10.1371/journal.pone.0208509

**Published:** 2018-12-27

**Authors:** Ce Shang, Xuening Wang, Frank J. Chaloupka

**Affiliations:** 1 Oklahoma Tobacco Research Center, Department of Pediatrics, Stephenson Cancer Center, University of Oklahoma Health Sciences Center, Oklahoma City, United States of America; 2 Department of Economics, University of Illinois at Chicago, Chicago, United States of America; 3 Division of Health Policy and Administration, School of Public Health, University of Illinois at Chicago, Chicago, United States of America; Centers for Disease Control and Prevention, UNITED STATES

## Abstract

Recent tobacco taxation research suggests that excise tax structure plays an important role in the effectiveness of increasing taxes in reducing consumption. However, evidence on excise tax structures of alcoholic beverages is scarce. We linked price variability measures for beer, wine, and liquor in the US derived using Economist Intelligence Unit city data from 2003 to 2016 with state-level excise tax structures from the Alcohol Policy Information System. Ordinary least squares (OLS) regressions were performed to assess the associations between excise tax structures and price variability, for beer, wine, and liquor (spirits), respectively. Results suggest that, compared with a specific excise beer tax structure based on volumes, a mixed structure with both specific and ad valorem components was associated with 38% (p≤0.01) greater beer price variability. In addition, a mixed excise tax structure for liquor was associated with 60–77% (p≤0.01) greater liquor price variability. However, these associations do not imply a causal link between tax structures and price variability. In summary, a mixed excise tax structure is associated with greater variability in beer and liquor prices, an indicator for tax avoidance opportunities. Future research is needed to identify the causal impact of tax structures on price variability.

## Introduction

Excessive drinking is a major cause for adverse health, economic, and behavior-related consequences.[1, 2] In the U.S., binge drinking leads to approximately 80,000 deaths and a cost of $224 billion annually.[3, 4] Among all policies aimed at reducing excessive drinking and related harm, increasing taxes is arguably the most effective intervention, and it is important to fully leverage its benefits.[5–9] However, tax avoidance behaviors may erode the effectiveness of such a policy as consumers can switch down to low-priced products, and thus are not responsive to tax and price increases by cutting back alcohol consumption or quitting excessive drinking. Moreover, vulnerable populations such as young and lower-income people are more likely to engage in tax avoidance and price minimization behaviors, undermining the effectiveness of increasing taxes and prices in reducing excessive drinking and related consequences among these populations. [10–15]

Recent studies on sin taxes (e.g., cigarette excise taxes) suggest that excise tax structure is an important factor associated with opportunities for tax avoidance and thus may affect the effectiveness of tax policies in reducing consumption.[16] Excise tax structures are defined by the tax base (quantities vs. values) and whether tax rates differ by product characteristics unrelated to the base, such as manufacturers’ size (uniform vs. tiered).[17] For example, excise alcohol taxes could be specific (i.e., volume-based or ethanol-based) or ad valorem (i.e., value-based) or a mix of both. Recent studies show that a combination of these taxes has more predictive power of prices, than a single type of taxes. [17, 18] Excise alcohol taxes could also vary by beverage types (i.e., beer vs. wine vs. liquor) and by consumption locations (i.e., off premise vs. on premise), but tiered rates do not apply within a beverage type or a consumption location in the US.[19]

Compared to a volume-based excise tax structure, ad valorem and mixed structures are considered more complicated because each different product may bear a different amount of taxes. The more complicated a tax structure is, the more opportunities there are for manufacturers to strategically differentiate brands and price levels, and for consumers to switch to cheaper products if taxes and prices were increased.[16, 20–22] A growing number of studies provided empirical evidence that more complicated cigarette tax structurers are associated with greater price variability—a key measure for tax avoidance opportunities. [23–25] However, such empirical evidence for the association between alcohol tax structure and price variability is lacking.

Unlike cigarette tax structures, which levy only specific excise taxes across all states in the U.S., alcohol excise tax structures differ by states. Some states levy only volume-based specific taxes while the rest levy a mixed structure with both ad valorem and volume-based taxes. [18, 26, 27] In many states, excise tax structure further varies by alcoholic beverage types and by premises. For example, Texas imposes both ad valorem and specific excise taxes on alcoholic beverages sold on premises but not on beverages sold off premises. Nonetheless, tax rates are uniform within the beverage-premise category in each state. Understanding how local alcohol excise tax structures are associated with price variability and tax avoidance opportunities thus is critical to informing and shaping future US alcohol taxation policies. As of January 2015, 19 out of 50 states allow localities to impose alcohol taxes, which presents an important opportunity for local actions to reduce excessive drinking and its burdens. [28]

This study is expected to provide the first assessment of alcoholic beverage price variability between states with different tax structures. Specifically, we examined state-level excise tax structures for beer, wine, and liquor and how they are associated with the respective price variability. Given that greater price variability is associated with more tax avoidance opportunities, the results will shed light on future studies on how to improve the effectiveness of alcohol taxation policies.

## Materials and methods

### Economist intelligence unit (EIU) city data

The EIU city data collected prices of more than 160 services and products including alcoholic beverages from supermarkets and mid-priced stores in 140 cities in 92 countries from 1990 to 2016. The data collection took place twice a year and the accuracy and consistency of data were checked by the EIU editorial team. Additionally, to insure the consistency over time and across geographical locations, prices of the same or similar brands were collected by the EIU for each product. Specifically for alcoholic beverages, average prices of one local brand beer, one top quality beer, one common table wine, one fine quality wine, and one superior quality wine were reported. For each product, prices were collected from two types of stores: supermarkets and mid-priced stores. Therefore, a total of four price points per year were available for beer in each city (i.e., local brand form supermarkets, local brand from mid-priced stores, top quality from supermarkets, and top quality from mid-priced stores), whereas six price points were available for wine (i.e., common table from supermarkets, common table from mid-priced stores, final quality from supermarkets, final quality from mid-priced stores, superior quality from supermarkets, and superior quality from mid-priced stores). The liquor category includes Cognac (French VSOP), Gin (Gilbey’s or equivalent), Liqueur Cointreau, and Scotch whisky six years old. For each of the above subtypes of liquor, one average price from supermarkets and one average price from mid-priced stores were reported. The EIU US data contain the prices of the above products from 16 cities in 15 states: Los Angeles (California), San Francisco (California), Miami (Florida), Atlanta (Georgia), Honolulu (Hawaii), Chicago (Illinois), Boston (Maryland), Detroit (Michigan), New York City (New York), Cleveland (Ohio), Pittsburgh (Pennsylvania), Seattle (Washington), Washington DC, Lexington (Kentucky), Minneapolis (Minnesota), and Houston (Texas).

### Alcohol policy information system (APIS)

The National Institute on Alcohol Abuse and Alcoholism’s (NIAAA) Alcohol Policy Information System (APIS) provides detailed information on alcohol-related policies and policy changes at the state level for beer (5% alcohol), wine (12% alcohol) and liquor (40% alcohol) in the U.S. from 2003–2016. APIS reports tax information for each of the three beverage types and the precise dates when tax rates changed. The following information is available in the APIS system: whether a state is a control state over sales that set shelf or minimum prices; [29] specific excise tax rates per gallon; and wholesale and retail ad valorem excise tax rates. Some states may levy special alcohol sales taxes in lieu of general sales taxes. Under such circumstances, ad valorem excise rates taking account of special sales taxes were considered as de facto ad valorem excise rates. The above information was further broken down for excise taxes on-premises and off-premises, respectively.

### Constructs

#### Price variability measures

We constructed price variability measures at the city-year level for each of the three beverage types: Beer, wine, and liquor. For beer, four price points (one local brand and one top quality brand from supermarkets and mid-priced stores) were available for each city in each year. For wine, six price data points were available in every city-year cell (one common table wine, one fine quality wine, and one superior quality wine, from supermarkets and mid-priced store). For each subtype of liquor (i.e., Cognac, Gin, Liqueur Cointreau, and Scotch whisky six years old), two data points were available, one from supermarkets and one from mid-priced stores.

Furthermore, beer prices were reported in different volumes. Local brands were reported on a per-liter basis, whereas top quality ones were reported on a per 330-ml basis. Therefore, we standardized beer prices to prices per liter. Wine prices were reported in the same volume (750 ml), as were Liquor prices (700 ml).

Respectively for each of the three beverage types, price variability was measured using a ratio of price range to average prices. These ratio measures are similar to other ratios that have been used in the literature examining price variability, such as a ratio of interquartile range to median or a ratio of price range to the lowest price. [16,23] The advantage of using ratios to measure price variability instead of interquartile range or variance, is that they are not affected by inflation and changes in the cost of living over time. Specifically, for beer and wine that have four to six price points in each city-year cell, we first ranked prices and then calculated the variability measure as the ratio of the difference between the highest and the lowest prices to the average of two middle prices ([maximum-minimum]÷middle price). For liquor, since there are two price points for each subtype (i.e., Cognac, Gin, Liqueur Cointreau, and Scotch whisky six years old), we first calculated, at the subtype level, the ratio of the price difference to the average price ([higher-lower]÷mean price). In the next step, we constructed the ratio measure for liquor as the mean of the four subtype ratios. We also constructed an alternative price variability measure for liquor using the largest ratio among the four subtypes.

#### Excise tax structures

During the study period, while all non-control states imposed specific excise taxes on the three alcoholic beverages, some states also imposed ad valorem excise taxes. Therefore, two forms of excise tax structures existed: specific only and a mixed structure of both specific and ad valorem components. The goal of the analyses thereby was to assess how a mixed tax structure, compared to a specific tax only structure, was associated with price variability.

Based on the tax information from APIS, we constructed yearly excise tax structure dummies (0 = a specific-only structure and 1 = a mixed structure) for each of the three beverage types. Furthermore, because EIU prices were collected from stores, only off-premise taxes were used to identify excise tax structures. In addition, some states had state monopolies in the retail of one or more beverage types. As a result, we also constructed a dummy for control states (1 = control states and 0 = non-control states) for each beverage type. Although several states changed tax rates during the study period, the change of excise tax structures were rare. On 12/8/2011, Washington State changed from a state monopoly (i.e., control state) to a specific-only tax structure in beer and wine sales, and to a mixed tax structure in liquor sales. Other than this change, tax structures were consistent over the study period.

### Demographic confounders

The demographic confounders considered in this study includes state-level unemployment rates from the Bureau of Labor Statistics, state-level median age, proportion of population aged 18–24, and per capita income from the US Census Bureau. EIU also reports city-level disposable income of a married person with two children as a percentage of a local-currency salary roughly equivalent to US $60,000, which was taken as a confounder in this study.

### Analysis

We linked price variability measures for beer, wine, and liquor at the city-year level with corresponding state-level excise tax structures using year and state identifiers. First, we used boxplot to evaluate the average price distribution for the period of 2012–2016 (after the structural change of Washington) by tax structures. Next, ordinary linear regressions (OLS) were used to assess the association between tax structures and price variability for each of the three alcoholic beverage types.

A total of three different specifications were examined. We started with a simple specification by regressing price variability outcomes on excise tax structures (a dummy of a mixed structure with a specific-only tax structure or control states as the reference group) and a dummy of control states without controlling for other confounders. In the second specification, we controlled for year fixed effects. In the third specification, we controlled for state and city demographic characteristics in addition to year fixed effects. We did not control for state fixed effects because of lack of changes in tax structurers over time. This choice was also supported by variance-inflation-factors that suggest high collinearity among state fixed effects, year fixed effects, and excise tax structures. Wild bootstrapped standard errors adjusting for clustering at the state level were estimated using Stata command “clustse” with 400 replications. Analyses were carried out using Stata 15 (StataCorp, Texas, USA).

## Results

In Table 1, we reported states’ control status and excise tax structures from 2003 to 2016 for beer, wine, and liquor, respectively. For beer taxes, Washington State was a control state until December 8th, 2011, and has applied specific taxes since then. Three states (Washington DC, Kentucky, and Minnesota) implemented a mixed excise tax structure on beer purchases off-premises with both specific and ad valorem excises. The remaining 11 states in the study applied only specific excises off-premises during the study period. For excise taxes on wine, Pennsylvania has been a control state for the entire study period. Similar to beer taxes, Washington State turned from a control state to a specific-only excise tax structure on December 8th, 2011. Three states (Washington DC, Kentucky, and Minnesota) adopted a mixed excise tax structure on wine with both specific and ad valorem taxes. The rest of the states applied only specific excise taxes. Finally, three states, Michigan, Ohio, and Pennsylvania, were control states for liquor. Washington State turned from a control state to a state with a mixed excise tax structure of liquor on December 8th, 2011. Washington DC, Kentucky, and Minnesota had a mixed excise tax structure for liquor and the other seven states had a specific-only structure for the entire study period.

**Table 1:** Excise tax structure by state.

Tax Structure	States (N = 15)	Cities (N = 16)
**Beer**		
Specific only	CA, FL, GA, HI, IL, MA, MI, NY, OH, PA,TX, WA (as of 2016-WA was a control state prior to 12/8/2011)	Los Angeles, San Francisco, Miami, Atlanta, Honolulu, Chicago, Boston, Detroit, New York City, Cleveland, Pittsburgh, Seattle, Houston
Mixed (specific and ad Valorem)	DC, KY, MN	Washington DC, Lexington, Minneapolis
Control State	WA (On 12/8/2011, Washington became a non-control state with specific taxation)	Seattle
**Wine**		
Specific only	CA, FL, GA, HI, IL, MA, MI, NY, OH,TX, WA (as of 2016- WA was a control state prior to 12/8/2011)	Los Angeles, San Francisco, Miami, Atlanta, Honolulu, Chicago, Boston, Detroit, New York City, Cleveland, Seattle, Houston
Mixed (specific and ad Valorem)	DC, KY, MN	Washington DC, Lexington, Minneapolis
Control State	PA, WA (On 12/8/2011, Washington became a non-control state with specific taxation)	Pittsburgh, Seattle
**Liquor**		
Specific only	CA, FL, GA, HI, IL, MA, NY, TX	Los Angeles, San Francisco, Miami, Atlanta, Honolulu, Chicago, Boston, Houston, New York City
Mixed (specific and ad Valorem)	DC, KY, MN, WA (as of 2016- WA was a control state prior to 12/8/2011)	Washington DC, Lexington, Minneapolis, Seattle
Control State	MI, OH, PA, WA (On 12/8/2011, Washington became a non-control state with both specific and Ad Valorem taxation)	Detroit, Cleveland, Pittsburgh, Seattle

**Fig 1:**
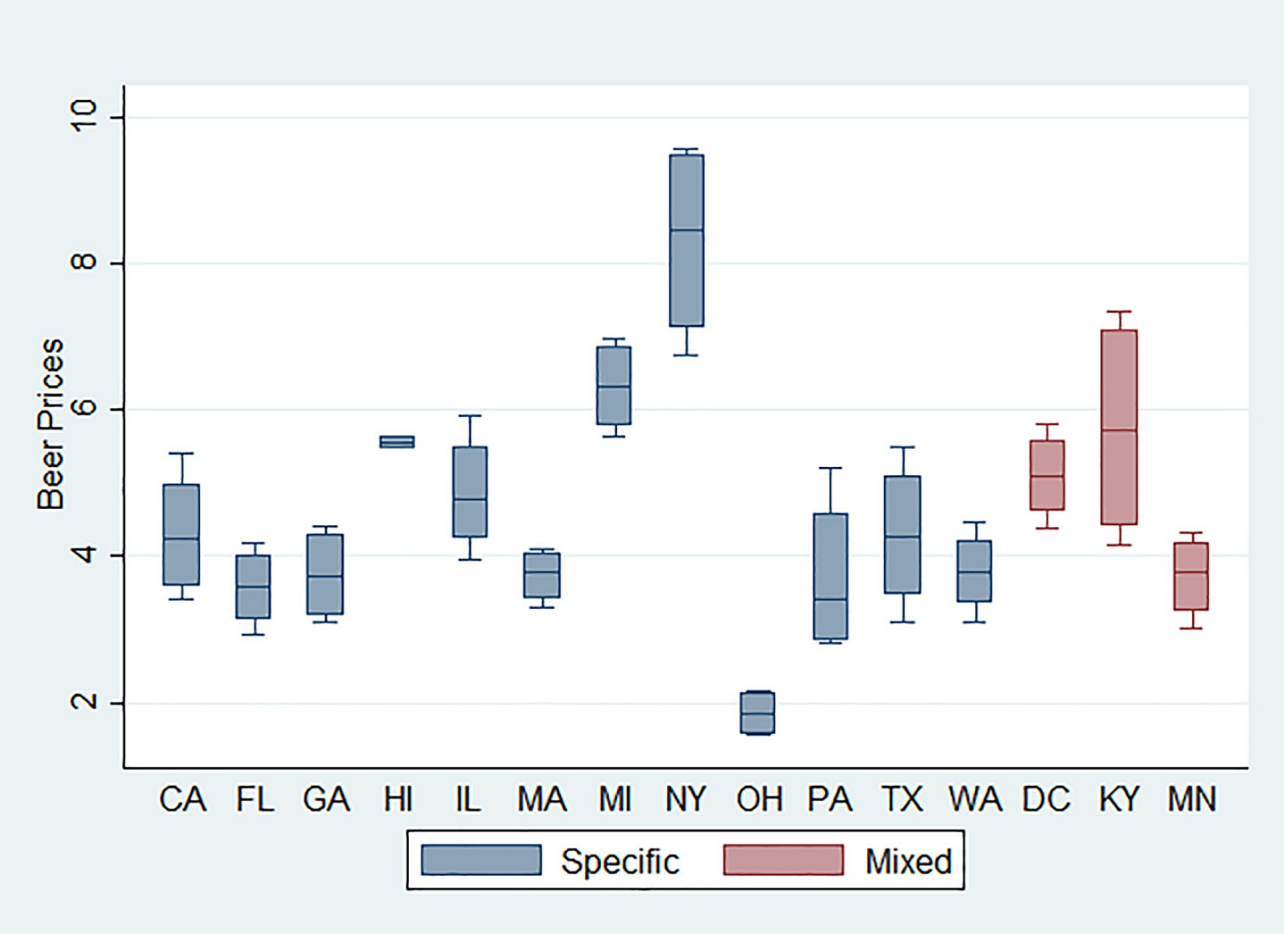
Boxplot of beer price distribution by state, 2012–2016.

**Fig 2:**
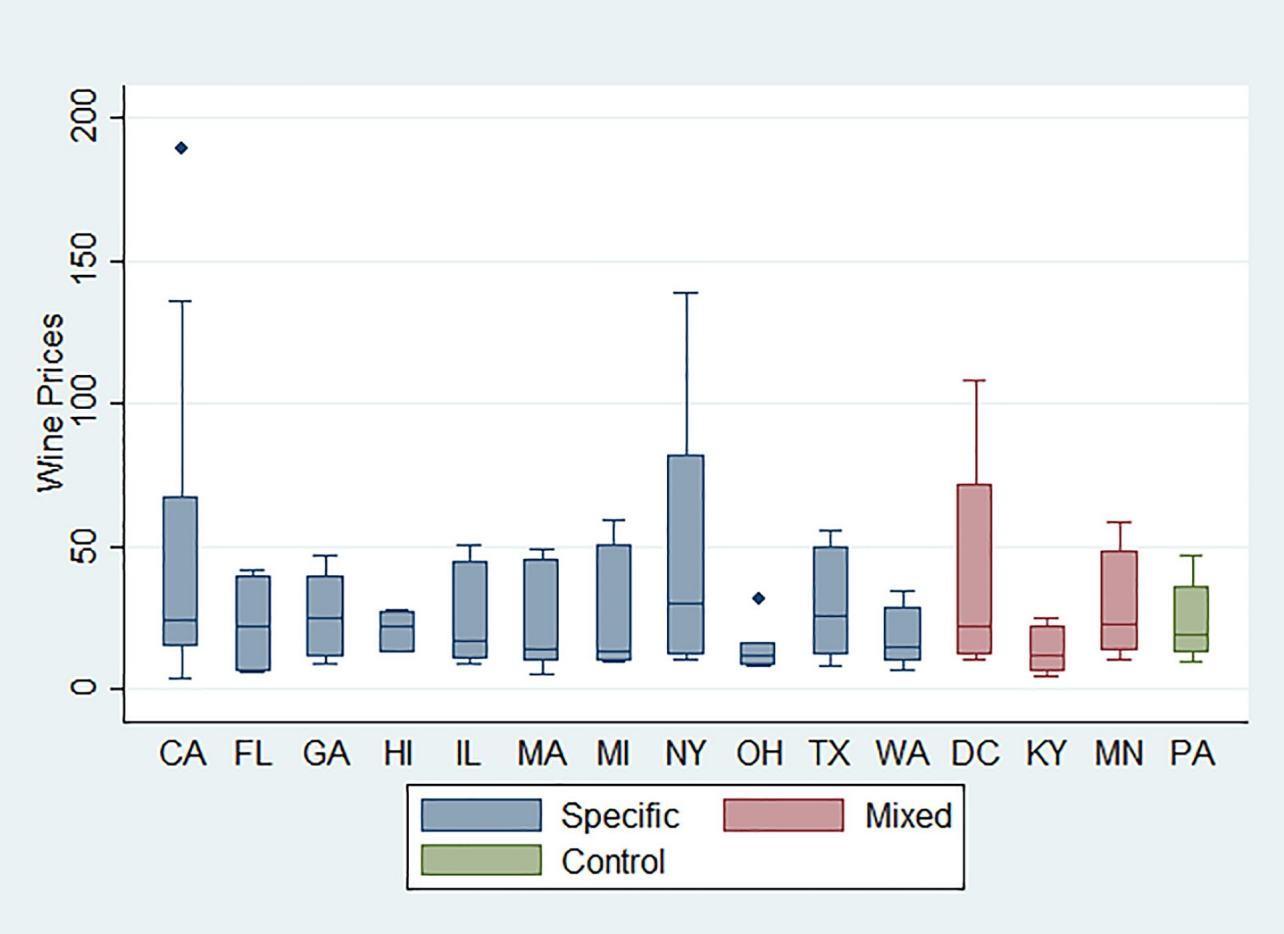
Boxplot of wine price distribution by state, 2012–2016.

**Fig 3:**
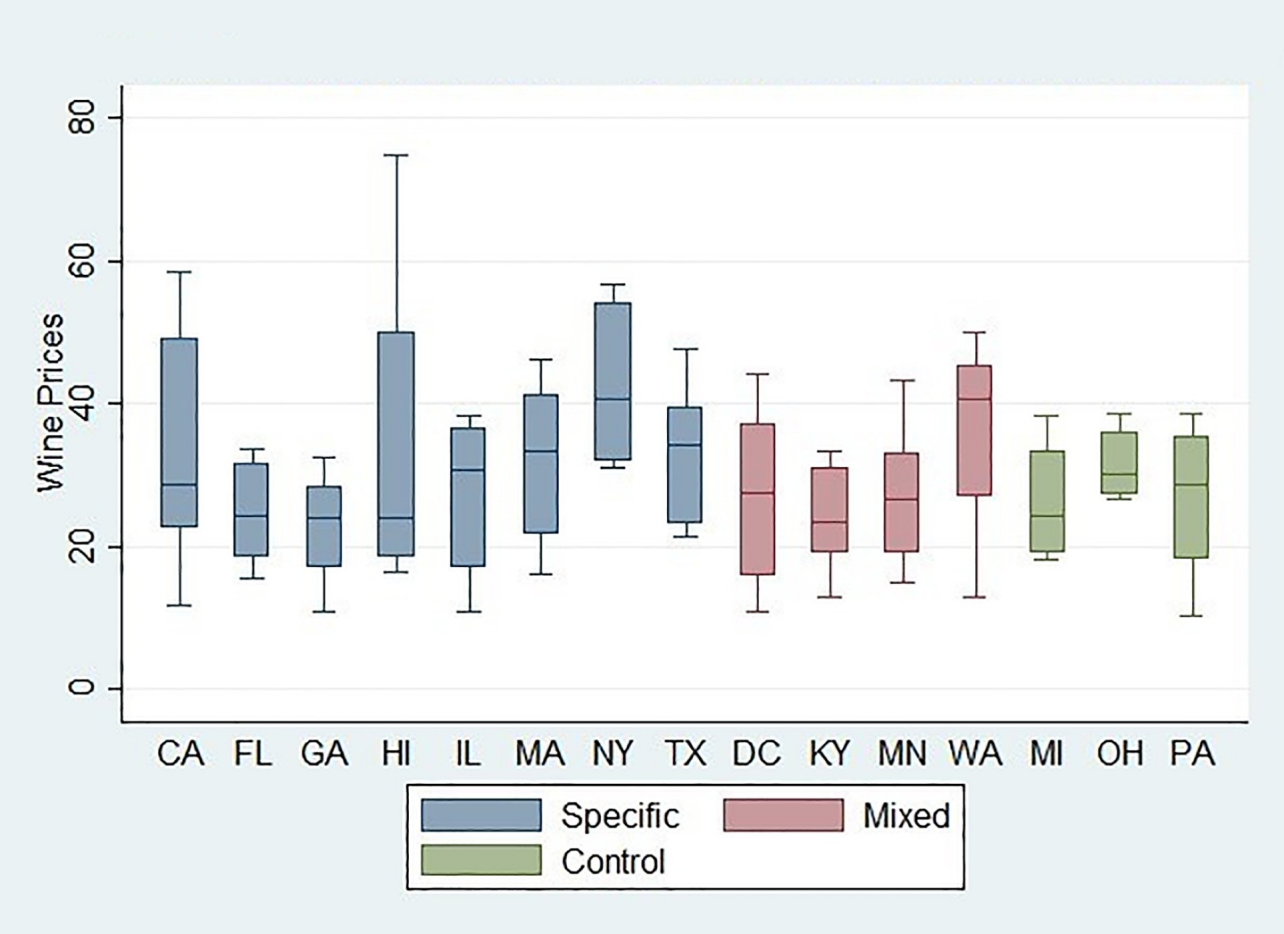
Boxplot of liquor price distribution by state, 2012–2016.

In Fig 1–3 we show the price distributions of the three beverages by excise tax structures. The boxplot of beer price distribution illustrates that price variability differs markedly by states. However, despite a similar price gap between higher- and lower- priced beers, as a state with the mixed tax structure, Kentucky had a lower average price than New York where only specific taxes were applied. This suggests a greater price variability for Kentucky than for New York. In addition, none of the states that had a mixed tax structure showed a narrow distribution, whereas some states with a specific-only excise tax structure, such as Hawaii, showed a narrow distribution. For wine, some states with a specific-only excise structure presented a narrow price distribution, such as Ohio and Hawaii. No clear patterns were found for liquor when comparing price distributions between a mixed structure and a specific-only structure.

Table 2 reports the summary statistics for the regression analyses. The price variability was 0.42 for beer, 1.72 for wine, and 0.15 for liquor (0.29 if measured as the maximum ratio among four liquor subtypes). For beer, 78% of the sample had a specific-only excise tax structure, 19% had a mixed excise tax structure, which has both specific and ad valorem components, and 4% had a state monopoly in beer sales. For wine, 71% had a specific-only excise tax structure, 19% had a mixed structure, and 10% had a state monopoly in wine sales. For Liquor, 56% had a specific-only excise tax structure, 21% had a mixed structure, and 22% had a state monopoly in sales. The average state-level median age was 37 years old and 10% of the population were young adults aged 18–25. Per capita income was $28,439 and the unemployment rate was 7%. The average city-level disposable income of a married person with two children was 80% of a local-currency salary, roughly equivalent to US $60,000.

**Table 2:** Summary statistics of price variability, tax structure and other covariates (N = 224).

Variables	Mean (%)	Standard Deviation
**Beer**		
Specific only	77.7%	
Mixed (specific and ad Valorem)	18.8%	
Control state	3.6%	
Price variability	0.42	0.21
Specific only (N = 174)	0.40	
Mixed (N-42)	0.47	
Control states (N-8)	0.54	
**Wine**		
Specific only	71.4%	
Mixed (specific and ad Valorem)	18.8%	
Control state	9.8%	
Price variability	1.72	0.47
Specific only (N = 160)	1.71	
Mixed (N = 42)	1.87	
Control States (N = 22)	1.47	
**Liquor**		
Specific only	56.3%	
Mixed (specific and ad Valorem)	21.4%	
Control state	22.3%	
Price variability		
Average across four types	0.15	0.11
Specific only (N = 125)	0.14	
Mixed (N = 48)	0.23	
Control states (N = 50)	0.10	
Maximum among four types	0.29	0.20
Specific only (N = 125)	0.28	
Mixed (N = 48)	0.40	
Control states (N = 50)	0.22	
**State-Level Control Variables**		
Median age	37.13	2.09
Percentage of population age 18–24	10%	0.9%
Unemployment rate	6.7%	2.1%
Per Capita Income ($)	28,439	5,103
**City-level Control Variables**		
Disposable income as a percentage of salary roughly equivalent to US$60,000	78.8%	4.5%

Table 3 presents the results from the regression analyses. We found that, compared to a specific-only beer excise tax structure, a mixed structure was associated with 0.16 (p≤0.05) greater beer price variability, after controlling for demographic confounders. The association between wine tax structures and price variability were not statistically significant. Compared to a specific-only liquor excise tax structure, a mixed tax structure was associated with 0.12 (p≤0.01) greater average price variability of the four liquor subtypes, and with 0.17 (p≤0.05) maximum price variability among the four subtypes. If we compare the associations to the average price variability for each beverage type, a mixed beer excise tax structure was associated with 38% greater beer price variability, whereas a mixed liquor excise tax structure was associated with 60–77% greater liquor price variability.

**Table 3:** The effect of the tax structure on the price variability of alcohol (N = 224).

Variables	(1)	(2)	(3)
**Beer**			
Specific only-omitted category			
Mixed	0.076 [-0.148,0.304]	0.075 [-0.151,0.3]	0.162** [0.073,0.251]
**Year fixed effects**	No	Yes	Yes
**State/city demographics**	No	No	Yes
**Wine**			
Specific only-omitted category			
Mixed	0.157 [-0.166,0.486]	0.157 [-0.165, 0.487]	0.081 [-0.282, 0.427]
**Year fixed effects**	No	Yes	Yes
**State/city demographics**	No	No	Yes
**Liquor-Average measure**			
Specific only-omitted category			
Mixed	0.089 [0.005,0.173]	0.090*** [0.007,0.172]	0.116*** [0.071,0.157]
**Liquor-Maximum measure**			
Specific only-omitted category			
Mixed	0.123 [-0/037, 0.286]	0.126 [-0.03, 0.286)	0.173** [0.098,0.254]
**Year fixed effects**	No	Yes	Yes
**State/city demographics**	No	No	Yes

Notes

* significant at 10%

** significant at 5%

*** significant at 1%.

All regression also controlled for a dummy for control states. Wild bootstrapped 95% CI adjusting for clustering the state level are reported in parentheses. When standard errors were adjusted for clustering at the state level, estimates in Column 3 were significant at 1% level for beer and liquor. Demographic control variables are state-level median age, percentage of population aged 18–24, per capita income, and unemployment rate, and city-level disposable income as a percentage of salary roughly equivalent to US$60,000.

## Discussion

We found that states with a mixed excise tax structure for beer and liquor had significantly greater price variability compared with states with a specific-only tax structure. Because greater price variability implies more opportunities for consumers to avoid increasing taxes by switching down to lower-priced products, this finding suggests that more tax avoidance opportunities may exist in states that apply a mixed excise tax structure on beer and liquor. As a result, there may be an association between excise tax structures of alcoholic beverages and the effectiveness of increasing taxes in curbing excessive drinking.

This study further adds to the literature that examines both specific and ad valorem excise taxes. In the U.S., a handful of recent studies assessed a combination of ad valorem excises, volume-based specific excises, and sales taxes of alcoholic beverages.[17, 18] Compared to studies that only assess volume-based excise taxes, this more comprehensive measure of combined taxes predicts more variation of alcohol prices. The price elasticity estimated using the combined taxes was greater than previous estimates, suggesting that consumers are more responsive to alcohol taxes and prices than previously estimated.[17] Our findings expand this line of research by showing that, although ad valorem excise taxes are an important component of the taxation system, they may be associated with greater price variability and thus with more tax avoidance opportunities.

We found that a mixed beer excise tax structure was associated with 38% greater beer price variability, whereas a mixed liquor excise tax structure was associated with 60–77% greater liquor price variability. However, wine excise tax structure was not significantly associated with price variability. This may be because the pricing strategy for wine is different from that for other alcoholic beverages; when the origins of the wine, instead of the qualities, plays an important role in pricing. [30] As a result, excise taxes may play a limited role in price distribution.

Although we found that a mixed excise structure is associated with more price variability, ad valorem taxes may have an advantage over specific taxes because they rise proportionally with the product costs, and thereby are less subject to the erosion of inflation over time. One recent study shows that the inflation-adjusted specific excise taxes in the US have been declining over time across wine, beer, and liquor.[31]] As a result, ad valorem taxes may be more effective in raising prices if specific taxes are not increased frequently enough to keep pace with inflation. Nevertheless, excise taxes on alcoholic beverages in the US are over-shifted to prices—a $1 increase in taxes would lead to a more than $1 increase in prices. Therefore, as a policy to reduce excessive drinking, increasing excise taxes has a significant public health impact.[32] More discussion is warranted to improve the effectiveness of taxation policies, including excise tax structures.

There are some limitations of the study. First, other than Washington state, none of the states in the study sample changed excise tax structures over time. Therefore, we could not employ a difference-in-difference model to identify the causal impact of excise tax structures on the price variability of alcoholic beverages. Second, there are limited price data points in the EIU data, which may underestimate the price variability of alcoholic beverages. Third, we only had price data from a selected sample of cities where population density is high. Therefore, the results are not nationally representative. In addition, relatively few states imposed a mixed structure, so the effects may have been driven by the factors related to alcoholic beverages in these few states that applied a mixed excise tax structure. Last, although price variability is the primary outcome of this study, tax structure may also impact average prices as tobacco tax structure literature suggests.[16] That is, average prices may be higher in a simple taxation system, such as a specific uniform system, compared with other taxation systems. The previous study also suggests that manufacturers’ market power may play a significant role in how taxes are passed to price distribution, including price variability. Future studies may explore simultaneously how excise tax structures and manufacturers’ market power together influence price distribution. Last, states that have a mixed excise structure may change their reliance on the specific and ad valorem components (e.g., an increase in ad valorem excise rates would increase the reliance on the ad valorem component), which may ultimately impact price variability. Future studies are needed to examine this additional relationship.

Nonetheless, we provided important empirical evidence that a mixed excise tax structure for beer and liquor is significantly associated with greater price variability. Therefore, alcohol excise tax structures may be associated with the effectiveness of alcohol taxes in reducing excessive drinking. Future research may employ changes in tax structures over a long period to assess the causal impact of tax structures on price variability. In addition, as tax structures vary by alcoholic beverage types, future research is needed to understand how taxation policies impact the relationships among different alcoholic beverage types (e.g., switching), an important factor related to the effectiveness of such policies in reducing excessive drinking.

## Conclusions

This study examined the association between excise tax structures of alcoholic beverages and price variability for three beverage types: beer, wine, and liquor. Results show that in the U.S., a mixed excise structure with both specific and ad valorem taxes, compared with a simple specific-only excise tax based on volumes, is associated with greater price variability, and thus with more opportunities for tax avoidance. This evidence suggests that excise tax structures may be associated with the effectiveness of increasing taxes or prices in reducing excessive drinking.

## References

[1] Corrao G, Bagnardi V, Zambon A, La Vecchia C. A meta-analysis of alcohol consumption and the risk of 15 diseases. Prev Med. 2004;38(5):613–9. 10.1016/j.ypmed.2003.11.027 . 15066364

[2] Rehm J, Mathers C, Popova S, Thavorncharoensap M, Teerawattananon Y, Patra J. Global burden of disease and injury and economic cost attributable to alcohol use and alcohol-use disorders. Lancet. 2009;373(9682):2223–33. 10.1016/S0140-6736(09)60746-7 . 19560604

[3] Bouchery EE, Harwood HJ, Sacks JJ, Simon CJ, Brewer RD. Economic Costs of Excessive Alcohol Consumption in the U.S., 2006. American Journal of Preventive Medicine. 2011;41(5):516–24. 10.1016/j.amepre.2011.06.045. 22011424

[4] Stahre M, Roeber J, Kanny D, Brewer RD, Zhang X. Contribution of Excessive Alcohol Consumption to Deaths and Years of Potential Life Lost in the United States. Preventing Chronic Disease. 2014;11:E109 10.5888/pcd11.130293 24967831 PMC4075492

[5] Xu X, Chaloupka FJ. The effects of prices on alcohol use and its consequences. Alcohol Res Health. 2011;34(2):236–45. 22330223 PMC3860576

[6] Elder RW, Lawrence B, Ferguson A, Naimi TS, Brewer RD, Chattopadhyay SK, et al The effectiveness of tax policy interventions for reducing excessive alcohol consumption and related harms. Am J Prev Med. 2010;38(2):217–29. 10.1016/j.amepre.2009.11.005 20117579 PMC3735171

[7] Wagenaar AC, Salois MJ, Komro KA. Effects of beverage alcohol price and tax levels on drinking: a meta-analysis of 1003 estimates from 112 studies. Addiction. 2009;104(2):179–90. 10.1111/j.1360-0443.2008.02438.x . 19149811

[8] Chaloupka FJ, Grossman M, Saffer H. The effects of price on alcohol consumption and alcohol-related problems. Alcohol Res Health. 2002;26(1):22–34. . 12154648 PMC6683806

[9] Grossman M, Chaloupka FJ, Saffer H, Laixuthai A. Effects of alcohol price policy on youth: A summary of economic research. Journal of Research on Adolescence. 1994;4(2):347–64. 10.1207/s15327795jra0402_9

[10] Meier PS, Purshouse R, Brennan A. Policy options for alcohol price regulation: the importance of modelling population heterogeneity. Addiction. 2010;105(3):383–93. 10.1111/j.1360-0443.2009.02721.x . 19839965

[11] An R, Sturm R. Does the response to alcohol taxes differ across racial/ethnic groups? Some evidence from 1984–2009 Behavioral Risk Factor Surveillance System. J Ment Health Policy Econ. 2011;14(1):13–23. 21552394 PMC3089007

[12] Nelson JP. Gender differences in alcohol demand: a systematic review of the role of prices and taxes. Health Econ. 2014;23(10):1260–80. 10.1002/hec.2974 . 23868570

[13] Mulia N, Ye Y, Greenfield TK, Zemore SE. Disparities in alcohol-related problems among white, black, and Hispanic Americans. Alcohol Clin Exp Res. 2009;33(4):654–62. 10.1111/j.1530-0277.2008.00880.x 19183131 PMC2771773

[14] Witbrodt J, Mulia N, Zemore SE, Kerr WC. Racial/ethnic disparities in alcohol-related problems: differences by gender and level of heavy drinking. Alcohol Clin Exp Res. 2014;38(6):1662–70. 10.1111/acer.12398 24730475 PMC4047188

[15] Lange JE, Voas RB, Johnson MB. South of the border: a legal haven for underage drinking. Addiction. 2002;97(9):1195–203. . 12199835 10.1046/j.1360-0443.2002.00182.x

[16] Chaloupka FJIV, Peck R, Tauras JA, Xu X, Yurekli A. Cigarette Excise Taxation: The Impact of Tax Structure on Prices, Revenues, and Cigarette Smoking. National Bureau of Economic Research Working Paper Series. 2010;No. 16287. 10.3386/w16287

[17] Xuan Z, Chaloupka FJ, Blanchette JG, Nguyen TH, Heeren TC, Nelson TF, et al The relationship between alcohol taxes and binge drinking: evaluating new tax measures incorporating multiple tax and beverage types. Addiction. 2015;110(3):441–50. 10.1111/add.12818 25428795 PMC4441276

[18] Xuan Z, Blanchette JG, Nelson TF, Nguyen TH, Hadland SE, Oussayef NL, et al Youth Drinking in the United States: Relationships With Alcohol Policies and Adult Drinking. Pediatrics. 2015;136(1):18–27. Epub 2015/06/03. 10.1542/peds.2015-0537 26034246 PMC4485013

[19] Saffer H, Chaloupka F. Alcohol Tax Equalization and Social Costs. Eastern Economic Journal. 1994;20(1):33–43.

[20] Chaloupka FJ, Straif K, Leon ME. Effectiveness of tax and price policies in tobacco control. Tob Control. 2011;20(3):235–8. Epub 2010/12/01. 10.1136/tc.2010.039982 . 21115556

[21] Shang C, Lee HM, Chaloupka FJ, Fong GT, Thompson M, O’Connor RJ. Association between tax structure and cigarette consumption: findings from the International Tobacco Control Policy Evaluation (ITC) Project. Tobacco Control. 2018 10.1136/tobaccocontrol-2017-054160 29794232 PMC6702940

[22] Pesko MF, Licht AS, Kruger JM. Cigarette price minimization strategies in the United States: price reductions and responsiveness to excise taxes. Nicotine Tob Res. 2013;15(11):1858–66. Epub 2013/06/05. 10.1093/ntr/ntt068 23729501 PMC3790627

[23] Shang C, Chaloupka FJ, Zahra N, Fong GT. The distribution of cigarette prices under different tax structures: findings from the International Tobacco Control Policy Evaluation (ITC) Project. Tobacco Control. 2013 10.1136/tobaccocontrol-2013-050966 23792324 PMC4009360

[24] Shang C, Chaloupka FJ, Fong GT, Thompson M, O'Connor RJ. The association between tax structure and cigarette price variability: findings from the ITC Project. Tob Control. 2015;24 Suppl 3:iii88–iii93. 10.1136/tobaccocontrol-2014-051771 25855641 PMC4612523

[25] Chaloupka FJ, Kostova D, Shang C. Cigarette excise tax structure and cigarette prices: evidence from the global adult tobacco survey and the U.S. National Adult Tobacco Survey. Nicotine Tob Res. 2014;16 Suppl 1:S3–9. 10.1093/ntr/ntt121 . 23935181

[26] Alcohol Policy Information System (APIS) 2017.

[27] M. K. Improving the measurement of state alcohol taxes. 2012.

[28] Mosher JF, Adler SS, Pamukcu AM, Treffers RD. Review of State Laws Restricting Local Authority to Impose Alcohol Taxes in the United States. Journal of studies on alcohol and drugs. 2017;78(2):241–8. Epub 2017/03/21. 10.15288/jsad.2017.78.241 28317504 PMC5554105

[29] Siegel M, DeJong W, Albers AB, Naimi TS, Jernigan DH. Differences in liquor prices between control state-operated and license-state retail outlets in the United States. Addiction. 2013;108(2):339–47. Epub 2012/09/01. 10.1111/j.1360-0443.2012.04069.x 22934914 PMC3529794

[30] Scozzafava G, Boncinelli F, Contini C, Romano C, Gerini F, Casini L. Typical Vine or International Taste: Wine Consumers' Dilemma Between Beliefs and Preferences. Recent patents on food, nutrition & agriculture. 2016;8(1):31–8. Epub 2015/12/31. . 26715305 10.2174/2212798408666151230114115

[31] Naimi TS, Blanchette JG, Xuan Z, Chaloupka FJ. Erosion of State Alcohol Excise Taxes in the United States. Journal of studies on alcohol and drugs. 2018;79(1):43–8. Epub 2017/12/12. 10.15288/jsad.2018.79.43 29227230 PMC5894857

[32] Shrestha V, Markowitz S. THE PASS-THROUGH OF BEER TAXES TO PRICES: EVIDENCE FROM STATE AND FEDERAL TAX CHANGES. Economic Inquiry. 2016;54(4):1946–62. 10.1111/ecin.12343

